# Identification of Primary Metabolite Profiles Reveals Quality Characteristics of *Citrus maxima* ‘Shatian Yu’ from Different Origins

**DOI:** 10.3390/cimb46110764

**Published:** 2024-11-11

**Authors:** Yujiao Peng, Meixin Li, Fangfei Song, Shuilan Liu, Yuxiang Qin, Baoqing Hu, Xueyu Cui

**Affiliations:** 1 Guangxi Geographical Indication Crops Research Center of Big Data Mining and Experimental Engineering Technology, Nanning Normal University, Nanning 530001, China; 2 Key Laboratory of Beibu Gulf Environment Change and Resources Utilization of Ministry of Education, Nanning Normal University, Nanning 530001, China

**Keywords:** ‘Shatian Yu’, fructification, differential metabolites, production area evaluation, quality

## Abstract

*Citrus maxima* ‘Shatian Yu’ displays varying nutritional profiles influenced by its production area. This study evaluated pomelo fruits from four primary and one developing ‘Shatian Yu’ production area. Notably, ‘Shatian Yu’ from the Guilin area exhibited higher sugar and alcohol content, suggesting enhanced taste. Principal component analysis and OPLS-DA revealed significant metabolite differences among production areas. In Guilin, variations were observed in a few substances, including sugars, alcohols, and phenolic acids. When compared with Rong City, Guilin showed a decrease in four phenolic acids and an increase in three organic acids, eighteen amino acids, eighteen lipids, and one vitamin. This comprehensive analysis provides valuable insights for the development of ‘Shatian Yu’ cultivation, highlighting the impact of production areas on fruit quality.

## 1. Introduction

*Citrus maxima* is a widely cultivated fruit worldwide [1] and has been produced in China for more than 4000 years; in addition, several varieties of the fruit are available, and the fruit is a rich resource. Pomelo is a plant in the *Rutaceae citrus* subfamily and is among the main varieties. It exhibits a characteristic fragrance, has a high medicinal value, and is sweet and sour, cool and moist, rich in nutrients, and very popular among consumers [2,3]. The nutritional components of pomelo fruit have been widely reported and include sugar, vitamin C, flavonoids, phenolic acids, limonoids, coumarin, volatile substances, and other nutrients [4,5]. These substances can not only provide basic energy and nutrition for human life activities but also have an ability to treat various diseases, such as diabetes [6], osteoporosis [7], inflammation, and colon cancer [8]. In addition, pomelo contains elements such as potassium, which is beneficial to patients with hypertension, cardiovascular disease, cerebrovascular disease, and kidney disease [9].

Various techniques employed in the field of metabolomics have been used to study differences in the metabolic profiles of fruits with different origins. Nuclear magnetic resonance spectroscopy (NMRS) can be used to assess mixture components without isolating compounds. This is a nontargeted method that directly utilizes spectroscopic measurements [10]. For example, Dominique Rolin et al. analyzed the changes in metabolites (organic acids, sugars, and amino acids) during the development of cherry tomatoes [11]. Metabolomics involves high-throughput detection and data processing and aims at information modeling and system integration. It is a newly developed discipline following genomics and proteomics and is an important part of systems biology. Compounds can be qualitatively and quantitatively analyzed through metabolomics using a combination of analytical tools that separate compounds, such as gas chromatography (GC) or liquid chromatography (LC), with mass spectrometry (MS). For example, Rui Min Vivian Goh et al. analyzed the components in Shatian pomelo peel oil by gas chromatography combined with quadrupole time-of-flight mass spectrometry (GC-QTOF/MS) and nonvolatile compounds using liquid chromatography (LC)-QTOF/MS [12]. This approach has been used more in metabolomics research in recent years. Fruit quality is affected by many factors, such as the variety type, environmental conditions, and health status. Metabolomics is a key strategy used to resolve these issues. Xiao et al. used metabolomics analysis technology to characterize tomato fruit metabolism under different light qualities. Their research revealed that blue light is more beneficial to amino acid metabolism and the synthesis of secondary metabolites [13]. Mauro et al. found that season is a key factor affecting the accumulation of primary metabolites, such as amino acids and sugars, in *Malus domestica* and *Pyrus communis* fruits [14].

*C. maxima* ‘Shatian Yu’ is native to Shatian Village, Rong County, Guangxi Province, and has a long history of cultivation in Rong County. ‘Shatian Yu’ has been expanded to the Xunjiang, Longjiang, Guijiang, Lijiang, and Hongshui River Basins in Guangxi, and Guangdong, Hunan, Sichuan, Zhejiang, and Jiangxi have also been introduced successively. Different origins exert obvious effects on fruit metabolite composition and biological activity. For example, bilberries (*Vaccinium myrtillus* L.) grown in areas with high photosynthetically active radiation (PAR) contain higher levels of total sugars, anthocyanins, flavonols, and hydroxycinnamic acids and lower levels of organic acids [15]. Evaluating fruit components in different producing areas can help to provide more comprehensive knowledge on the role of environmental factors in the accumulation of primary and secondary metabolites. At the same time, excellent production areas can be discovered.

To the best of our knowledge, none of the literature has reported the differences in the material composition of ‘Shatian Yu’ fruits from different regions. Therefore, we compared the composition of ‘Shatian Yu’ fruit from four main producing areas and one developing area in China. This research will provide guidance for the selection of ‘Shatian Yu’ planting sites. Moreover, the results are valuable for determining the theoretical accumulation and attribution of functional components in ‘Shatian Yu’.

## 2. Materials and Methods

### 2.1. ‘Shatian Yu’ Fruit Harvesting

‘Shatian Yu’ from different origins was used as the test material. ‘Shatian Yu’ was picked in November 2021, and all picking was completed before November 10. The sampling locations (shown in Figure S1) were Shishan Town in Meizhou City (MZ, 24°26′51.2″ N, 116°5′9.2″ E), Guangdong Province; Xingping Town in Guilin County (GL, 24°46′50.8″ N, 110°29′32.1″ E), Guangxi Province; Ziliang Town in Rong City (RX, 22°51′41.4″ N, 110°33′13.3″ E), Guangxi Province; Cushijiang Town in Jiangyong City (JY, 25°5′5.3″ N, 110°59′36.5″ E), Hunan Province; and Linfeng Town, Changshou District (CS, 29°54′4.8″ N, 107°11′44.1″ E), Chongqing. RX is the traditional production area of ‘Shatian Yu’ and is the most famous area. In terms of climate, all production areas are dominated by tropical and subtropical monsoon climates, which are generally characterized by high temperatures and rain in summer and mild temperatures and little rain in winter. In this climatic environment, most of the soil is red soil. Three orchards were selected for each point, and each orchard was a mature orchard that had produced fruit for over 10 years. Three fruits of similar size (1 kg) and fruit shape were collected from the orchards.

### 2.2. Sample Pretreatment

The ‘Shatian Yu’ fruits from different sources were peeled and the pulp near the equator of the fruit was obtained from each pomelo field. The same amount of pulp was taken from three fruits and mixed into one sample, and three samples from the same area were used as three replicates.

### 2.3. Metabolite Analysis Using UPLC–MS/MS

Biological samples (shown in Section 2.2 Sample pretreatment) were vacuum freeze-dried in a freeze dryer (Scientz-100F, Scientz, Ningbo, China) and ground (30 Hz, 1.5 min) to a powder using a grinder (MM 400, Retsch, Haan, Germany). We weighed 100 mg of the powder, dissolved it in 1.2 mL of 70% methanol extract, and vortexed it every 30 min for 30 s each time, for a total of 6 times. Subsequently, samples were placed in a 4 °C refrigerator overnight. After the samples were removed and centrifuged (12,000 rpm, 10 min), the supernatant was aspirated, and the samples were filtered with a microporous membrane (0.22 μm pore size) and stored in a sample vial for UPLC–MS/MS analysis.

The column was equilibrated with mobile phase A (0.1% formic acid ultrapure aqueous solution). Metabolites were eluted using a linear gradient of mobile phase B (acetonitrile with 0.1% formic acid). At 0.00 min, the proportion of phase B was 5%, and within 9.00 min, the proportion of phase B increased linearly to 95% and remained at 95% for 1 min. At 10.00–11.10 min, the proportion of phase B decreased to 5% and was balanced at 5% for 14 min. The column temperature was set at 40 °C. The flow rate was 0.35 mL/min, the column temperature was 40 °C, and the injection volume was 4 μL.

Linear ion trap (LIT) and triple quadrupole (QQQ) scans were acquired on a triple quadrupole linear ion trap mass spectrometer (Q TRAP), AB4500 Q TRAP UPLC/MS/MS system(AB SCIEX, Singapore City, Singapore). The system is equipped with an ESI Turbo ion spray interface, which can be controlled by Analyst 1.6.3 software (AB Sciex, Shanghai, China) to run positive and negative ion modes. The ESI source operating parameters were as follows: ion source, turbo spray; source temperature, 550 °C; ion spray voltage (IS), 5500 V (positive ion mode)/−4500 V (negative ion mode); ion source gas I (GSI), gas II (GSII), and curtain gas (CUR) at 50, 60, and 25.0 psi, respectively. The collision-induced ionization parameter was set to high. Instrument tuning and mass calibration were performed with 10 and 100 μmol/L polypropylene glycol solutions in QQQ and LIT modes, respectively. QQQ scans were performed using multiple reaction monitoring (MRM) mode with the collision gas (nitrogen) set to medium. The substance was characterized according to the secondary spectrum information and the isotope signal. The repetitive signal containing K^+^, Na^+^, and NH_4_^+^, and the repetitive signal of the fragment ion of other larger-molecular-weight substances were removed during the analysis.

Metabolite quantification was performed using multiple reaction monitoring (MRM) analysis. After the mass spectrometry data of metabolites from different samples was obtained, the peak areas of all mass spectrum peaks were integrated, and the mass spectrum peaks of the same metabolite in different samples were integrated and corrected [16].

### 2.4. Multivariate Pattern Analysis and Differential Metabolites Selected

Unsupervised principal component analysis (PCA) was performed by the statistics function prcomp within R (www.r-project.org, version 4.0). The data were unit variance-scaled before unsupervised PCA. The R package MetaboAnalystR was used for orthogonal partial least squares discriminant analysis (OPLS-DA) analysis [17]. The screening threshold for differential metabolites was *VIP* > 1 and *p* value < 0.05.

### 2.5. Metabolic Pathway Analysis

Identified metabolites were annotated using the KEGG Compound database (http://www.kegg.jp/kegg/compound/) (accessed on 4 November 2024), and annotated metabolites were then mapped to the KEGG Pathway database (http://www.kegg.jp/kegg/pathway.html) (accessed on 4 November 2024) [18]. Pathways mapping significantly regulated metabolites were then fed into metabolite set enrichment analysis (MSEA), and their significance was determined by hypergeometric test *p* values.

### 2.6. Correlation Analysis

The top fifty differential compounds with *VIP* > 1 between groups were extracted for correlation analysis. The Pearson correlation coefficients (PCCs) between samples were calculated by the cor function in the R package ComplexHeatmap and presented as only heatmaps.

## 3. Results

### 3.1. Comprehensive Metabolite Profiling of ‘Shatian Yu’ Across Different Production Areas

**Figure 1:**
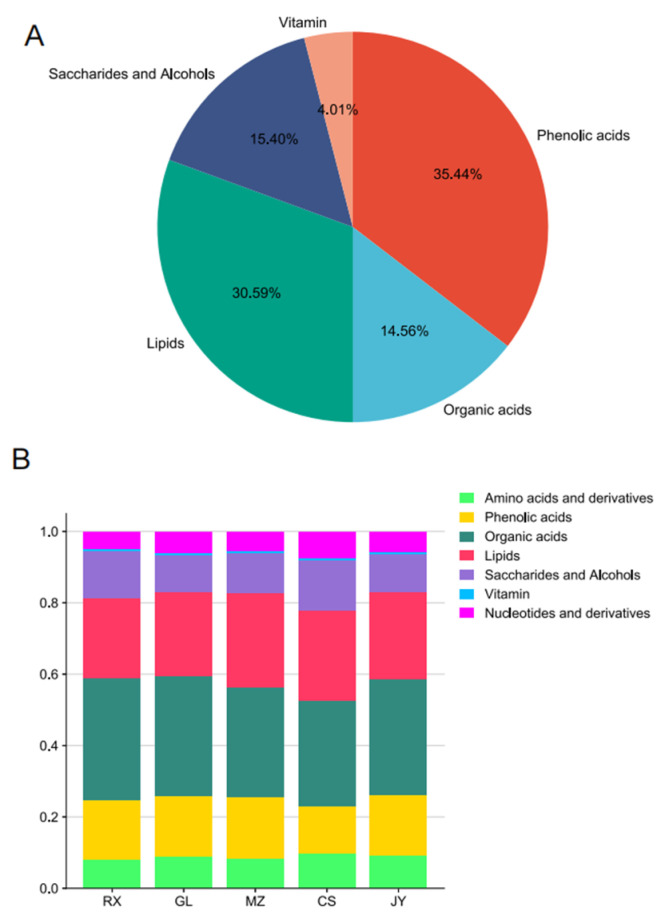
Metabolite characteristics of ‘Shatian Yu’ from different producing areas. (**A**) The types and proportions of substances contained in ‘Shatian Yu’. (**B**) The relative content of primary metabolites in ‘Shatian Yu’ from different origins. Different colors represent different species of matter. The abscissa is the ‘Shatian Yu’ from different origins, and the ordinate is the relative content percentage of the substance.

UPLC–MS/MS was used to detect the metabolites of ‘Shatian Yu’ plants with different origins. A total of 623 metabolites were detected in ‘Shatian Yu’ (Supplementary Table S1 and Figure 1A), including 101 kinds of amino acids and their derivatives, 168 kinds of phenolic acids, 68 kinds of organic acids, 72 kinds of sugars and alcohols, 17 kinds of vitamins, and 143 kinds of lipids (Supplementary Table S1 and Figure 1A). All metabolite species overlapped in the four samples, indicating that the differences between the pomelo samples were mainly reflected in the content rather than the type of metabolites. We further conducted an analysis on the relative content of primary metabolites in pomelo fruit with different origins, and pomelo contains higher quantities of lipids, amino acids, and derivatives, followed by organic acids, sugars, alcohols, phenolic acids, and vitamins, which are low (Figure 1A). The relative content of phenolic acids in pomelo from CS and JY production areas was higher than that of GL, while the relative content of phenolic acids in pomelo from RX and MZ production areas was lower than that of GL. The relative content of organic acids in MZ was the highest, followed by JY, RX, and GL, and the relative content of organic acids in CS was the smallest. The relative content of lipids was highest in RX and GL pomelo. Except for the RX production area, the relative contents of amino acids and their derivatives in the pomelo of MZ, CS, and JY were higher than those of GL. The relative content of sugar and alcohol in pomelo from the GL production area was the highest, and the relative content of vitamins was the lowest (Figure 1B).

### 3.2. Differential Metabolite Analysis in ‘Shatian Yu’ from Various Production Areas

**Figure 2:**
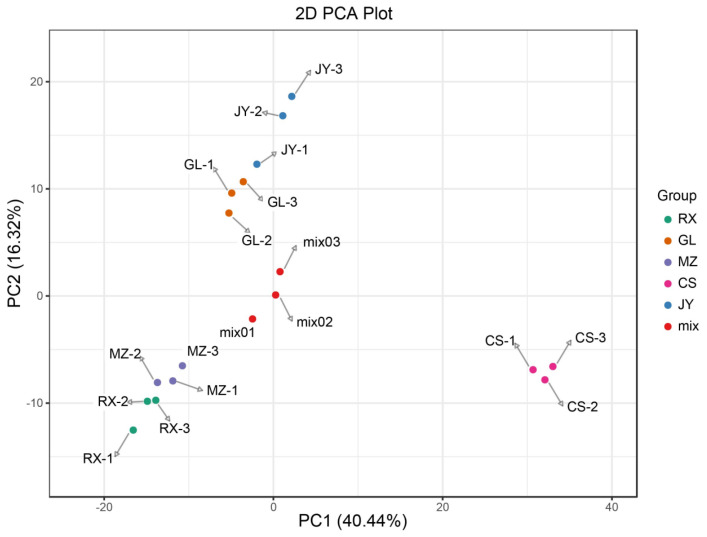
PCA of ‘Shatian Yu’ from different production areas.

**Figure 3:**
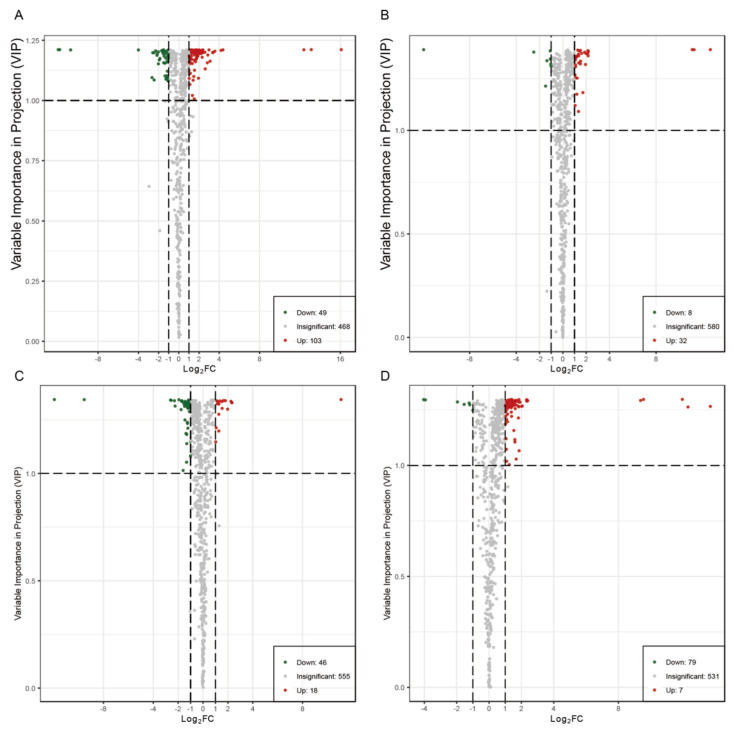
Differential metabolite analysis. (**A**) GL vs. CS; (**B**) GL vs. JY; (**C**) GL vs. MZ; (**D**) RX vs. GL. Among them, the green points represent the downregulated differential metabolites, the red points represent the upregulated differential metabolites, and the gray points represent the metabolites detected but not significantly different. The abscissa represents the logarithmic value of the relative content difference in a certain metabolite in the two groups of samples (log_2_FC). The vertical axis indicates the *VIP* value.

PCA score plots are good methods for describing differences between or within groups. As shown in Figure 2, the five groups of samples were well aggregated in different regions, indicating that the repeatability of the experiment is good. The contribution rates of PC1 and PC2 to sample separation were 40.44% and 16.32%, respectively. Guilin is not the main production area of pomelo. To explore the influence of the main production area on the metabolites of ‘Shatian Yu’, an OPLS-DA model was established to determine the metabolic differences in ‘Shatian Yu’ from the main production area and Guilin. As shown in Supplementary Figure S2, the score plots of the four models indicated good separation between the different groups. According to high predictability (Q2) and further verification by permutation tests (Supplementary Figure S3), an OPLS-DA model with better predictability and reliability was established. In this study, differential metabolites were screened in OPLS-DA according to *VIP* > 1 and *p* value < 0.05 in the independent sample *t* test, and the results are shown in Table S2. Screening results were illustrated using volcano diagrams (Figure 3) and Venn diagrams (Supplementary Figure S4). In total, 152 differentially produced metabolites were identified between GL and CS (103 upregulated and 49 downregulated) (Figure 3A); 40 differentially produced metabolites were found between GL and JY (32 upregulated and 8 downregulated) (Figure 3B); 64 differentially produced metabolites were found between GL and MZ (18 upregulated and 46 downregulated) (Figure 3C); and 86 differentially produced metabolites were identified between RX and GL (79 upregulated and 7 downregulated) (Figure 3D). Compared with Guilin, the four main production areas shared four differential metabolites (Supplementary Figure S4). Compared with GL, the relative contents of dambonitol and 6-O-acetylarbutin in ‘Shatian Yu’ from other producing areas were generally upregulated, while the content of dehydrodiconiferyl alcohol was generally downregulated. Sinapoylglucuronic acid was significantly upregulated in RX, MZ, and JY and showed the opposite trend in CS.

### 3.3. Comparative Analysis of Differential Metabolites in ‘Shatian Yu’ from Different Origins

**Figure 4:**
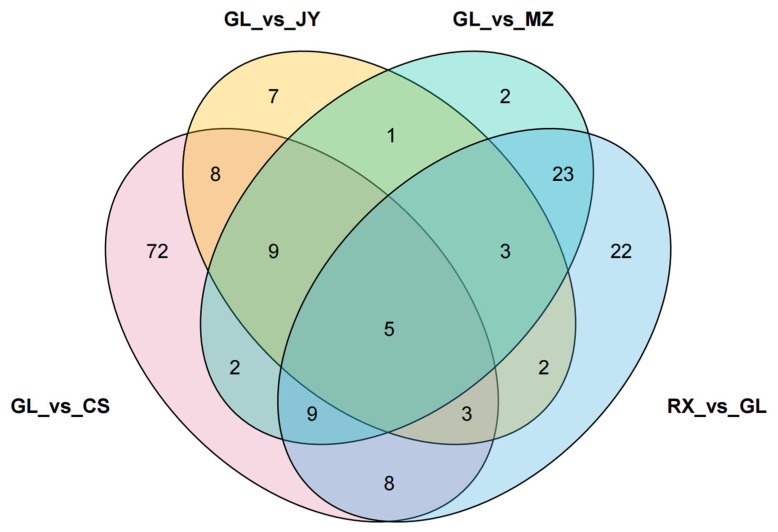
Venn analysis of substances among pomelo from different origins. Numbers indicate the quantity of unique and shared substances.

The specific differences in substances among pomelo with different origins are shown in Figure 4 and Supplementary Table S2 (an asterisk (*) following a compound name denotes the existence of structural isomers for that particular compound).

Compared with GL, 33 phenolic acids in pomelo from CS were significantly upregulated, including p-coumaric acid, caffeic aldehyde, caffeic acid, and ferulic acid. Twenty-eight kinds of phenolic acids of CS origin were significantly downregulated, including cinnamic acid, methyl caffeate, and alnusonol. The 11 kinds of phenolic acids in pomelo from JY were significantly higher than those from GL, including sinapyl alcohol, benzoylmalic acid, and tachioside. Six kinds of phenolic acids decreased significantly, including cinnamic acid, dehydrodiconiferyl alcohol, bis(p-coumaroyl)tartaric acid, and dehydrodiconiferyl alcohol-gamma’-O-glucoside. Compared with GL, six phenolic acids were significantly upregulated in MZ, including methyl caffeate, tachioside, tachoside and 6-O-acetylarbutin. Fourteen phenolic acids were significantly downregulated, including cinnamaldehyde, cinnamic acid, p-coumaryl alcohol, rhododendrol, 3-hydroxy-4-methoxybenzoic acid, vanillic acid, coniferaldehyde, 4-methoxyphenylpropionic acid, coniferyl alcohol, p-dimeric galloyl methyl ester, etc. Compared with GL, only four phenolic acids were significantly upregulated in RX, including 6-O-acetylarbutin, 5-glucosyloxy-2-hydroxybenzoic acid methyl ester, trihydroxycinnamoylquinic acid, and sinapoylglucuronic acid. In RX, 25 metabolites were significantly downregulated, including cinnamaldehyde, anthranilic acid, 4-aminobenzoic acid, 4-hydroxybenzoic acid, 2,5-dihydroxybenzaldehyde, protocatechualdehyde, protocatechualdehyde, p-coumaryl alcohol, 3,4-dihydroxybenzoic acid (protocatechuic acid)*, rhododendrol, vanillic acid, coniferaldehyde, coniferyl alcohol, ferulic acid*, isoferulic acid*, and sinapic acid.

Eight organic acids were significantly upregulated in pomelo originated from CS, including succinic semialdehyde, hydroxypyruvic acid, 3-guanidinopropionic acid, phenylpyruvic acid, dl-3-phenyllactic acid, 2-propylmalic acid*, 3-isopropylmalic acid*, and 2-isopropylmalic acid. Seven phenolic acids decreased significantly, including d-malic acid, l-malic acid, 2,3-dihydroxy-3-methylbutanoic acid, diethyl phosphate, and jasmonic acid. There was no significant difference in organic acid metabolites between pomelo from JY and GL. Two organic acids were upregulated, including 2-propylmalic acid* and 2,4-dichlorophenoxyacetic acid, and two organic acids were downregulated, including diethyl phosphate and jasmonic acid. Compared with GL, there was no difference in other organic acid components except diethyl phosphate, which was significantly downregulated. Only three organic acids were significantly downregulated in RX, including 2-picolinic acid, 2,2-dimethylsuccinic acid, and 2-isopropylmalic acid.

Nineteen amino acids were significantly upregulated in CS compared with GL, including L-ornithine, L-tyrosine, L-homocitrulline, L-tryptophan, L-leucyl-L-phenylalanine, and L-phenylalanyl-L-phenylalanine. Seven amino acids were significantly downregulated, including L-asparagine and L-aspartic acid. Six amino acids in pomelo grown in JY were significantly upregulated, including L-glutamic acid and L-arginine. Four amino acids in pomelo grown in MZ were significantly upregulated, including L-glutamic acid, L-threo-3-methylaspartic acid, N-α-acetyl-L-ornithine, and L-leucyl-L-phenylalanine. Seven amino acids were significantly upregulated, including L-ornithine, N-glycyl-L-leucine*, L-glycyl-L-isoleucine*, L-tyrosine methyl ester, etc. Eighteen amino acids in RX were significantly downregulated, including L-asparagine, L-ornithine, N-acetyl-L-threonine, 1-methylhistidine, and L-arginine.

Twelve lipids were significantly upregulated in CS compared with GL, including lysoPC 12:0, lysoPC 16:2, lysoPC 16:2 (2n isomer), lysoPC 19:2 (2n isomer), and lysoPC 19:1. Five substances were downregulated, including 3-dehydrosphinganine, 2r-hydroxyoctadecanoic acid, and ethyl 9-hydroxy-10,12-octadecadienoic acid. Five lipids were significantly upregulated in JY compared with GL, including (E)-linalool-1-oic acid, 9-oxo-10E,12Z-octadecadienoic acid, 9-Oxo-10E,12Z-octadecadienoic acid, and 9-hydroxy-12-oxo-10(E),15(Z)-octadecadienoic acid. Two substances were significantly downregulated, including heptadecanoic acid and petroselinic acid. Four substances were significantly upregulated in MZ compared with GL, including 17-hydroxylinolenic acid, 9(S), 12(S), 13(S)-trihydroxy-10(E)-octadecenoic acid, 9,10-Dihydroxy-12,13-epoxyoctadecanoic acid, and LysoPC 19:2(2n isomer). Ten substances were significantly downregulated, including 3-dehydrosphingosine, DL-2-hydroxystearic acid, 9-hydroxy-12-oxo-15(Z)-octadecenoic acid, (5S,8R,9Z,12Z)-5,8-dihydroxyoctadeca-9,12-dienoate, ethyl 9-hydroxy-10,12-octadecadienoic acid, 1-α-linolenic acid glyceride*, and lysophosphatidylglycerol 16:1. Eighteen substances were significantly downregulated in RX compared with GL, including 12-oxo-phytodienoic acid, 3-ehydrosphinganine, 1-oleoyl-sn-glycerol, lysoPG 16:1, and lysoPC 19:2.

Compared with GL, 14 sugars and alcohols were significantly upregulated in CS, including L-glucose, D-glucose, D-fructose, mannose, and D-sorbitol. One sugar and alcohol substance was significantly downregulated, which was 3-dehydro-L-threonic acid. Two sugars and alcohols were significantly upregulated in JY, including dambonitol and raffinose. Two substances were significantly upregulated in MZ compared to GL, including dambonitol and 1-(sn-glycero-3-phosphate)-1D-myo-inositol. One substance was significantly downregulated in MZ, including melezitose O-rhamnoside. In contrast to GL, RX contained a significantly upregulated substance, dambonitol. Compared with GL, two sugars and alcohols were significantly downregulated in MZ, which were ribulose-5-phosphate and D-Melezitose O-rhamnoside.

Compared with GL, one vitamin substance (nicotinamide) was significantly downregulated in CS. Two vitamins were significantly downregulated in MZ, nicotinic acid (vitamin B3) and pyridoxine. RX contained one vitamin that was significantly downregulated, which was pyridoxine.

### 3.4. KEGG Pathway Analysis of ‘Shatian Yu’ Metabolites

**Figure 5:**
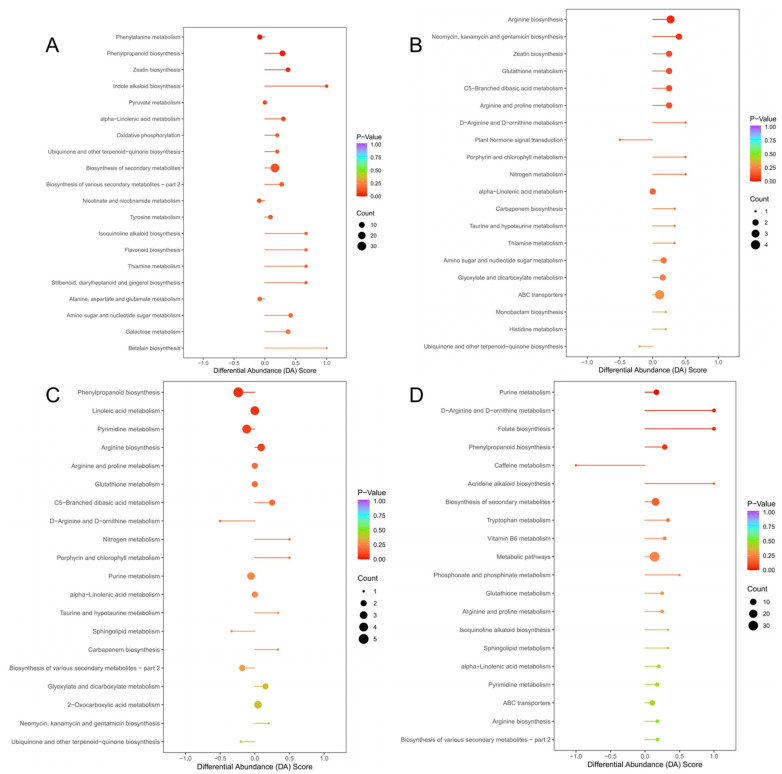
KEGG DA score of differential metabolites. (**A**) GL vs. CS; (**B**) GL vs. JY; (**C**) GL vs. MZ; (**D**) RX vs. GL. The ordinate indicates the name of the differential pathway, and the abscissa indicates the differential abundance score (DA score). The DA score reflects the overall change in all metabolites in the metabolic pathway. A score of 1 means that the expression trend is upregulated for all identified metabolites in this pathway, and −1 means that the expression trend is downregulated for all identified metabolites in this pathway. The length of the line segment represents the absolute value of the DA score, and the size of the dot following the line segment represents the number of differential metabolites in the pathway. The dots are distributed on the left side of the central axis, and the overall expression of the pathway is more likely to be downregulated when the line segment is longer. The dots are distributed on the right side of the central axis, and the overall expression of the pathway will more likely be upregulated when the line segment is long, and the larger the dots are, the more metabolites. The color of the line segments and dots reflects the *p* value. The closer the color is to red, the smaller the *p* value, and the closer the color is to purple, the larger the *p* value.

The KEGG database was used to analyze the metabolic pathway enrichment of these four groups of differential metabolites. The top 20 metabolic pathways are listed according to the *p* value in Supplementary Table S3. The most important metabolic pathways were further screened, and the results are displayed in differential abundance score plots. The DA score reflects the overall change in all metabolites in the metabolic pathway; the length of the line represents the absolute value of the DA score, and the size of the dot after the line represents the number of differential metabolites in the pathway. The line length and dot color reflect the *p* value size. Indole alkaloid biosynthesis, isoquinoline alkaloid biosynthesis, flavonoid biosynthesis, thiamine metabolism, stilbenoid, diarylheptanoid, and gingerol biosynthesis, and betalain biosynthesis were the main differential enrichment pathways of CS and GL (Figure 5). D-arginine and D-ornithine metabolism, porphyrin and chlorophyll metabolism, and nitrogen metabolism were the main differential metabolic enrichment pathways of JY, MZ, and GL. D-Arginine and D-ornithine metabolism, folate biosynthesis, caffeine metabolism, and acridone alkaloid biosynthesis were the main differential metabolic enrichment pathways of RX and GL.

### 3.5. Correlation Analysis of Differential Substances

**Figure 6:**
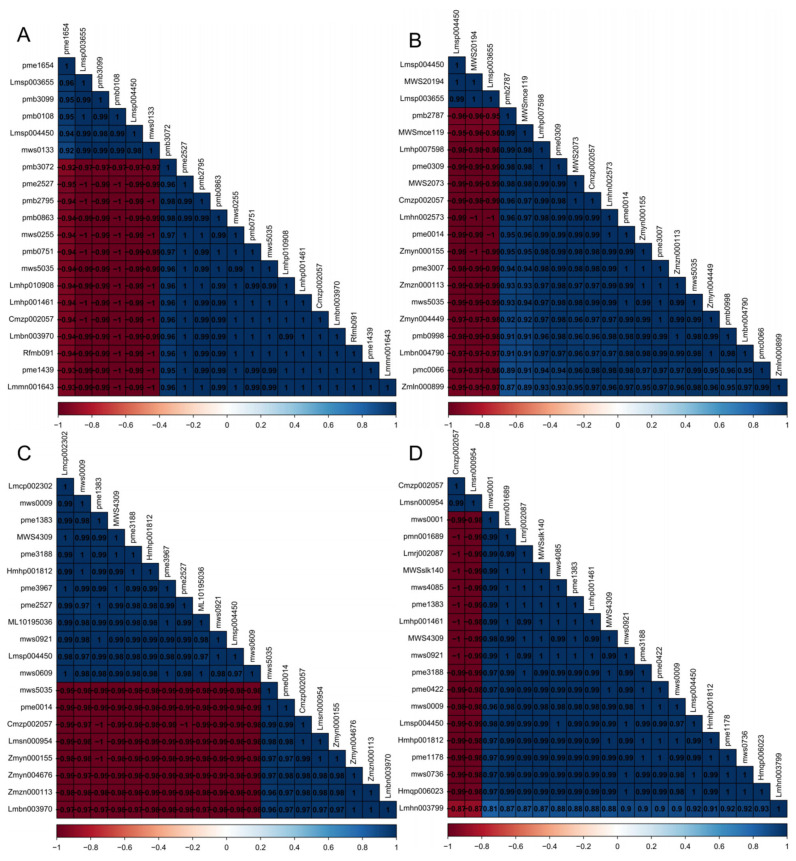
Differential substance correlation heatmap. (**A**) GL vs. CS; (**B**) GL vs. JY; (**C**) GL vs. MZ; (**D**) RX vs. GL. The corresponding square color between substances represents the degree of correlation. The darker the color is, the greater the correlation.

We performed a Pearson correlation analysis on the differential metabolites screened out by the top 20 *VIP* values, and the analysis results were displayed in the form of a heatmap, as shown in Figure 6, and the indexes that correspond to the compounds are shown in Table S4. Metabolite correlations often reveal the cooperativity of changes between metabolites. The synergistic change in the relative content of substances is consistent with the upstream and downstream relationship of substance synthesis to some extent. In GL vs. CS (Figure 5A), a total of 84 combinations of metabolites exhibited strong negative correlation patterns, primarily involving pme1654, Lmsp003655, pmb3099, pmb0108, Lmsp004450, and mws0133. Additionally, GL vs. CS displayed the best metabolite correlations among all production areas, as evidenced by the presence of numerous regions with a correlation value of 1, indicating a strong positive correlation among these metabolites. In GL vs. JY (Figure 5B), a total of 51 combinations of metabolites showed strong negative correlation patterns, mainly centered around Lmsp004450, MWS20194, and Lmsp003655. Notably, Lmsp004450 and Lmsp003655 also appeared in GL vs. CS. The majority of the remaining metabolites exhibited a relatively good positive correlation. In GL vs. MZ (Figure 5C), a total of 96 combinations of metabolites demonstrated strong negative correlation patterns. MZ exhibited the most prominent negative correlations among differentially expressed metabolites when compared to GL, primarily involving mws5035, pme0014, Cmzp002057, Lmsn000954, Zmyn000155, Zmyn004676, Zmzn000113, and Lmbn003970. Despite having the highest number of negatively correlated metabolites, GL_vs_MZ also showcased the strongest positive correlations among metabolites, with all correlation coefficients exceeding 0.95. In RX vs. GL (Figure 5D), only 36 combinations of metabolites exhibited negative correlations, all of which were associated with Cmzp002057 and lmsn000954. The remaining metabolites displayed positive correlations, with many correlation coefficients reaching 1, indicating a strong correlation. This indirectly suggests that the differences between Shatian Yu from the GL production area and the traditional RX production area are smaller compared to the differences with other production areas. Furthermore, there is a clear correlation between a large number of organic acids, phenolic acids, and amino acids.

### 3.6. Phenylpropanoid Pathway Analysis in ‘Shatian Yu’ from Different Regions

**Figure 7:**
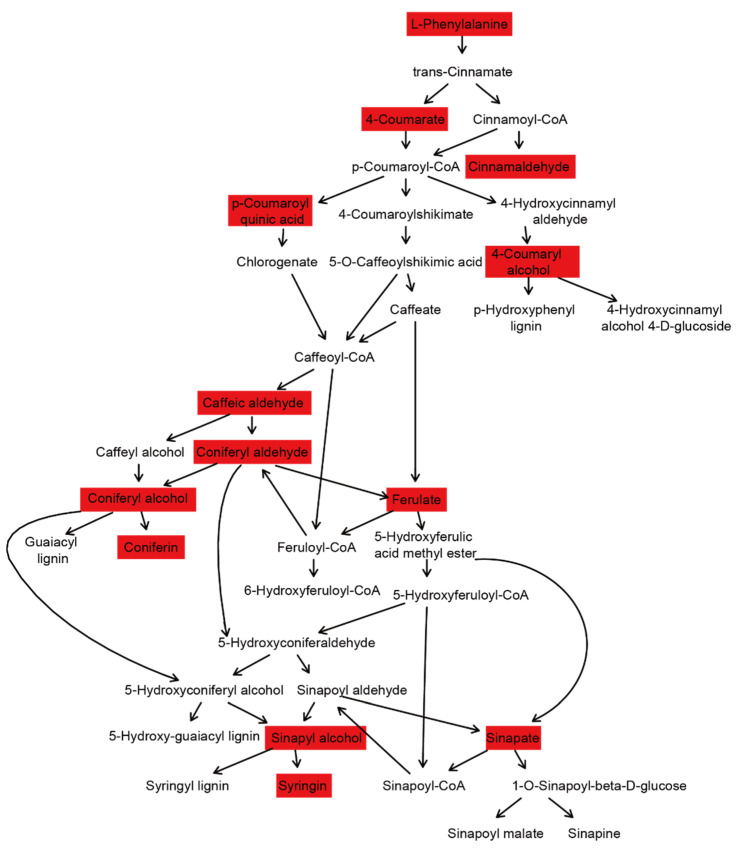
Phenylpropanoid metabolic pathway analysis. The metabolites detected in this study are set against a red box.

We matched the metabolites in ‘Shatian Yu’ to the phenylpane metabolic pathway, and the results showed that the 13 metabolites of ‘Shatian Yu’ were detected in the metabolic pathway (Figure 7). Matching substances include L-phenylalanine, 4-coumarate, cinnamaldehyde, p-coumaroyl quinic acid, 4-coumaryl alcohol, caffeic aldehyde, coniferyl aldehyde, coniferyl alcohol, ferulate, coniferin, sinapyl alcohol, sinapate, and syringin. The relative contents of cinnamaldehyde, p-coumaryl alcohol, and coniferaldehyde in GL grapefruit were significantly higher than those in MZ and RX grapefruit. In addition, GL also possessed higher contents of conferyl alcohol, ferulic acid*, and sinappic acid. The vast majority of metabolites of GL and JY in the phenylpropanoid pathway were not significant (Supplementary Table S2). GL utilizes a strong phenylpropanoid metabolic pathway, which may lead to more abundant secondary metabolites. However, some substances were not detected. Compared with GL, the metabolites involved in the pathway of JY were similar, indicating the potential of JY to produce effective secondary metabolites. In CS, two substances decreased and three substances increased, indicating that the environmental impact of this production area was different from that of the GL production area; however, it did not inhibit the production of beneficial substances but only fine-tuned the composition of metabolites. The most downregulated substances were MZ and RX, which indicated that the accumulation of secondary metabolites produced by the phenylpropanoid pathway was not good in these two production regions.

## 4. Discussion

Pomelo contains protein, fat, carbohydrates, and dietary fiber and is rich in vitamins and trace elements. The primary metabolites changed substantially in ‘Shatian Yu’ from different production areas with Guilin. Amino acids are very important components of food because they are the building blocks of proteins. They are divided into essential amino acids and nonessential amino acids. Essential amino acids need to be obtained from external sources, while nonessential amino acids are synthesized by the body [19]. Among plant foods, fruits are rich sources of various amino acids [20]. The total relative abundance of amino acids in CS, JY, and MZ was higher than that in GL (Figure 1B and Supplementary Table S1), indicating that GL may lead to less amino acid production compared to that of the remaining main producing areas. The relative content of amino acids and their derivatives in pomelo from GL was higher than that from RX (Figure 1B and Supplementary Table S1), which indicated that GL had a developmental value. L-Aspartic acid exhibits a concentration-dependent excitatory effect on brain slice metabolism [21]. L-Aspartate is metabolically converted to L-glutamine, L-arginine, and glutathione [22]. In addition to their anti-inflammatory and antioxidant properties, these amino acids prevent lipid peroxidation and improve liver microcirculation [23]. Our results showed that the L-asparagine and L-aspartic acid contents of GL were significantly higher than those of CS (Figure 3A and Supplementary Table S2). Glutamate is a major excitatory neurotransmitter [24,25]. Glu functions as a multifaceted metabolite and cellular signaling molecule within plant systems. The biosynthetic pathway of glutamate is intrinsically linked to primary nitrogen assimilation processes. Through the interconversion between glutamate and 2-oxoglutarate, glutamate metabolism is integrated with the tricarboxylic acid cycle, carbon metabolic pathways, and cellular energy generation mechanisms [26]. Endogenous L-glutamic acid plays an important role in alleviating anxiety, neurodegenerative diseases, and sleep disorders [24,25]. L-arginine (l-arg) is metabolized to nitric oxide (NO) by nitric oxide synthase NOS [27,28]. NO has been recognized as a critical signaling molecule in plants, playing diverse roles in plant developmental processes. Furthermore, NO regulates responses to both biotic and abiotic stresses, which involve extensive reprogramming of cellular metabolism [29]. The earliest abnormality of atherosclerosis is endothelial dysfunction [30]. NO modulates endothelial function and antiatherosclerotic properties [31]. Thrombolytic therapy combined with injection of L-arginine improves endothelial dysfunction in a porcine model [32]. This study found that the L-glutamic acid and L-arginine contents of GL were higher than those of JY (Figure 3B and Supplementary Table S2).

In plants, phenolic compounds not only protect plant cells from disease but also filter out toxic UV rays and protect fragile plant seeds until they germinate. When humans taste certain polyphenol-rich plant foods, especially fruits, these polyphenols become beneficial free radical scavengers [33]. They have been reported to perform various biological functions, including generating antioxidant, anti-inflammatory, anticancer, and antiaging effects [34]. Citrus plants contain a large number of phenolic compounds that are beneficial to human health [35]. Wang et al. detected different phenolic acids in a citrus peel: chlorogenic acid, p-coumaric acid, ferulic acid, sinapinic acid, and caffeic acid [36]. Our research found 168 phenolic acids in pomelo (Figure 1B and Supplementary Table S1). This reflects the nutritional value and health care value of pomelo. The total amount of phenolic acids in pomelo grown in GL was higher than that in RX and MZ, which indicated that pomelo in GL may produce more phenolic acids, which can provide more nutrition and help balance the human diet. Fraga et al. found that long-term intake of orange juice can reduce blood pressure and body fat, which is partly due to its phenolic acid components [37]. Scholars have reported that vanillic acid can inhibit the proliferation of various cancer cells, such as breast cancer, colon cancer, and lung cancer [38]. Vanillic acid exerts analgesic and anti-inflammatory effects that depend on the inhibition of neutrophils, oxidative stress, cytokines, and NF-κB channels [39]. Studies by Russo et al. showed that when the daily intake of hydroxybenzoic acid is high, the risk of prostate cancer becoming advanced is lowered [40]. In our study, we found that the relative content of vanillic acid in GL was significantly higher than that in RX and CS, while the relative content of hydroxybenzoic acid was significantly higher than that in RX, indicating the health care potential of GL (Figure 3D and Supplementary Table S2).

The acidity of citrus fruits is mainly due to the levels of citric and malic acids [41]. Our results showed that there was no significant difference in the citric acid levels of pomelo from GL and the main producing areas, but the malic acid levels were significantly higher compared with that of RX (Figure 3 and Supplementary Table S2). Jasmonic acid (JA) is a phytohormone that responds to biotic and abiotic stresses; in addition, JA is a signaling substance that regulates cellular defense responses [42,43,44]. We found that the JA content of GL was higher than that of CS and JY (Figure 3A,B and Supplementary Table S2). The role of the jasmonic acid signaling pathway in the fruit response to pathogen infection is well known. Examples include resistance of sweet cherries to Botrytis cinerea infection [45], resistance of Eriobotrya japonica fruit to anthracnose infection [46], and resistance of citrus fruit to Penicillium digitatum infection [47]. We hypothesize that fruit from the GL origin can be stored and transported more conveniently. Jasmonic acid and its derivatives (such as 7-isojasmonic acid, methyl jasmonate (MeJA), methyl dihydrojasmonate (MeDiJA), etc.) can be metabolically transformed into each other [48]. Previous studies have shown that methyl jasmonate exerts a therapeutic effect on mixed basal cell carcinoma [49]. Malignant melanoma is a serious skin cancer that originates in cells called melanoma. Jasmonic acid induces melanoma cell death [50]. In addition to organic acids, sugars are also important indicators to measure the quality of fruits, and they can change the taste of fruits. The study found that GL showed less of a difference than RX, JY, and MZ (Figure 1B and Figure 3 and Supplementary Table S1). CS achieved a higher relative content of various carbohydrates (Figure 3 and Supplementary Table S2). We hypothesize that pomelo from GL was of good quality, even though the taste of CS may be better.

Lipid compounds in plant fruits contain a large amount of energy, and some lipids have been reported to treat certain diseases. Glycerol phenylbutyrate can alleviate the condition of patients with urea cycle enzyme deficiency [51]. Glyceryl trinitrate is an effective drug for alleviating/treating tendinopathy [52] and strokes [53]. Our research shows that RX and GL have the highest proportion of lipid content, indicating that pomelo in GL production areas may have a more beneficial effect on health.

Phenylpropane metabolic pathways play an important role in plant responses to biotic and abiotic stresses [54,55,56]. Starting with L-phenylalanine, plants produce approximately 8000 compounds through complex chemical reactions [57]. The most important compounds in these reactions are flavonoids. They not only act as phytoprotectins and antioxidants in plant disease resistance but also play an important role in the treatment of human diseases, such as cardiovascular and cerebrovascular diseases, diabetes, and neurodegenerative diseases [58,59]. Our results show that there were more compounds in the phenylpropane pathway in GL (Figure 7 and Supplementary Table S2). This may indicate its flavonoid-producing potential. Flavonoids were not detected in this experiment.

In conclusion, our results indicate the good quality and health benefits of ‘Shatian Yu’ from GL, which may be an upcoming pomelo production area. Moreover, an additional area of research is to investigate whether climatic conditions and microbial communities also influence the quality of pomelo. This remains a direction for further exploration.

## 5. Conclusions

This study has identified that the primary metabolites in ‘Shatian Yu’ fruit are phenolic acids and lipids. We observed that the proportions of sugar, alcohol, and total lipids in pomelo from the GL region are higher compared to those from other production areas. Additionally, the content of phenolic acid and organic acid in fruits from GL is found to be at an intermediate level among the various origins. These findings establish GL as a crucial candidate area for the cultivation of ‘Shatian Yu’. Significantly, this research offers valuable insights and a solid reference for the high-quality cultivation of ‘Shatian Yu’, facilitating informed decisions in agricultural practices and production area development.

## Data Availability

Data are contained within the article or Supplementary Materials.

## Supplementary Materials

The following supporting information can be downloaded at https://www.mdpi.com/article/10.3390/cimb46110764/s1.
