# Supplementary material for: Identification of Primary Metabolite Profiles Reveals Quality Characteristics of Citrus maxima ‘Shatian Yu’ from Different Origins
Source: Curr Issues Mol Biol. 2024 Nov 11;46(11):12830–46. doi: 10.3390/cimb46110764 (PMC11593049; doi:10.3390/cimb46110764)
Supplement: Supplementary file 1 [file cimb-46-00764-s001.zip › Supplementary Figure edited.pdf]

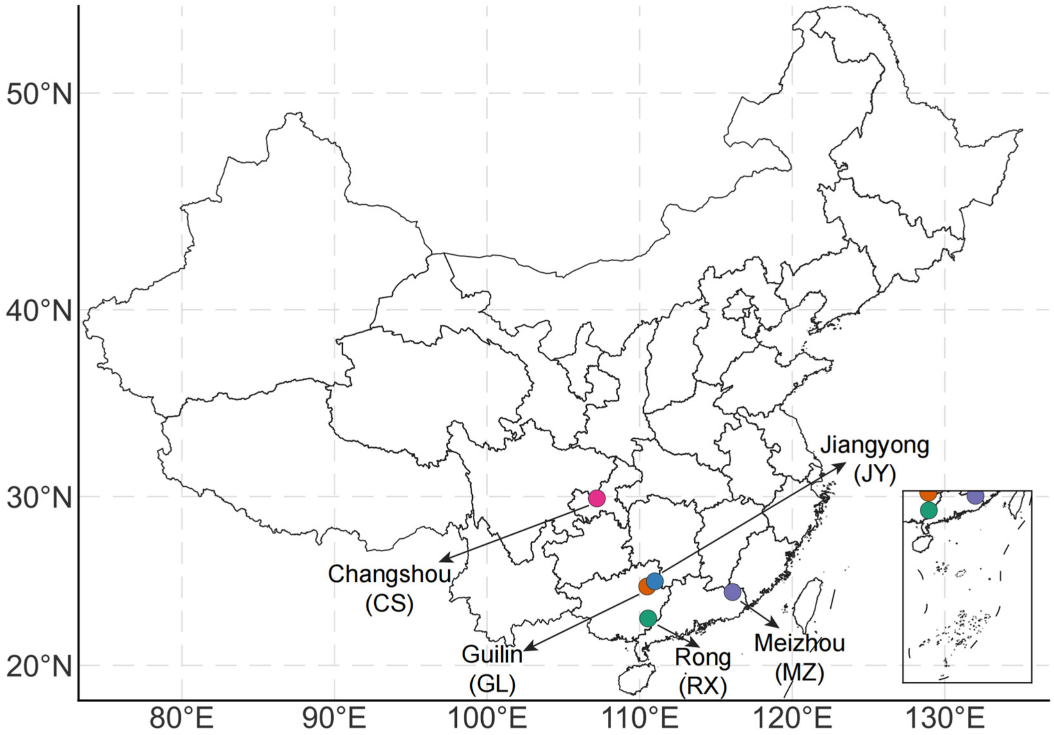

Figure S1. Sampling site distribution map.

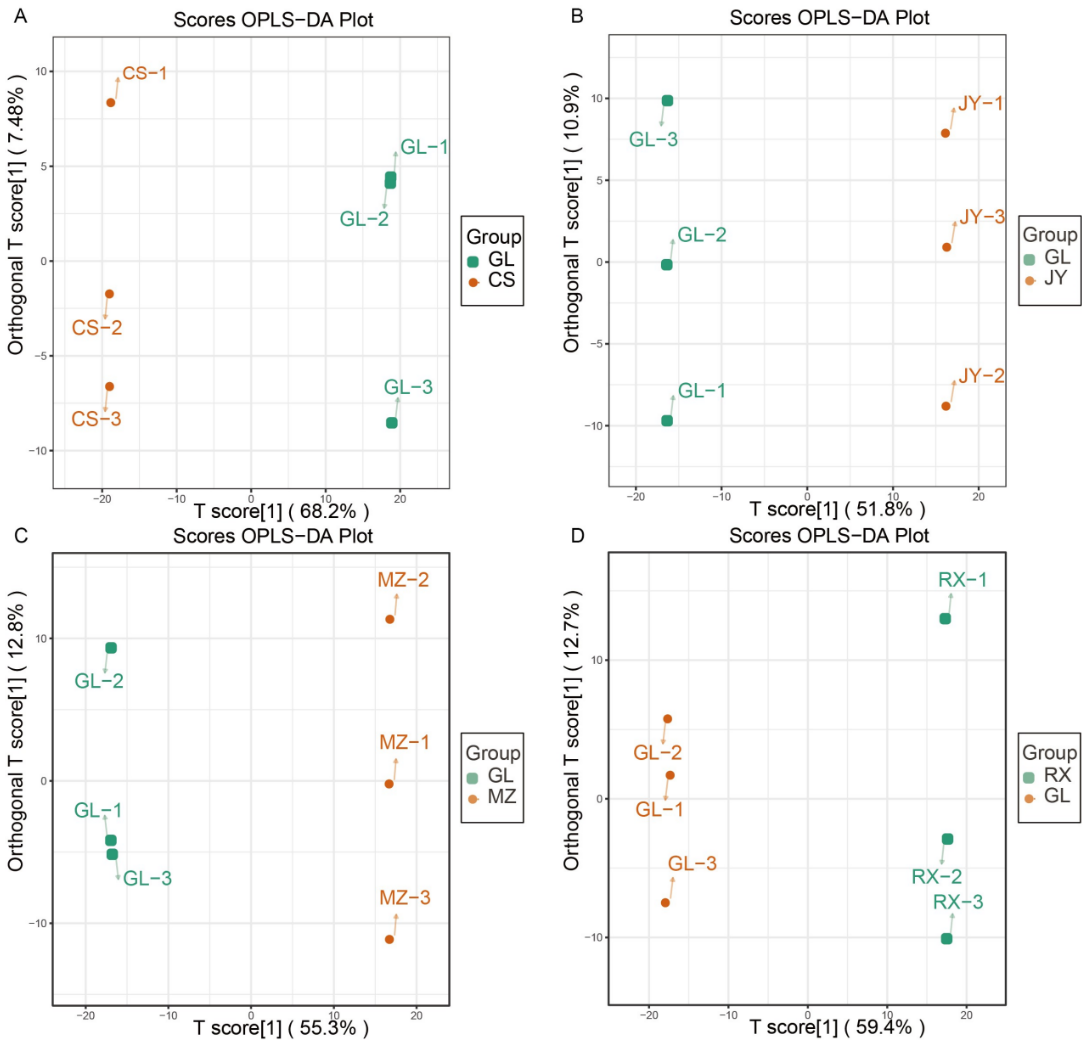

Figure S2. OPLS-DA score map. A. GL vs. CS; B. GL vs. JY; C. GL vs. MZ; D. RX vs. GL.

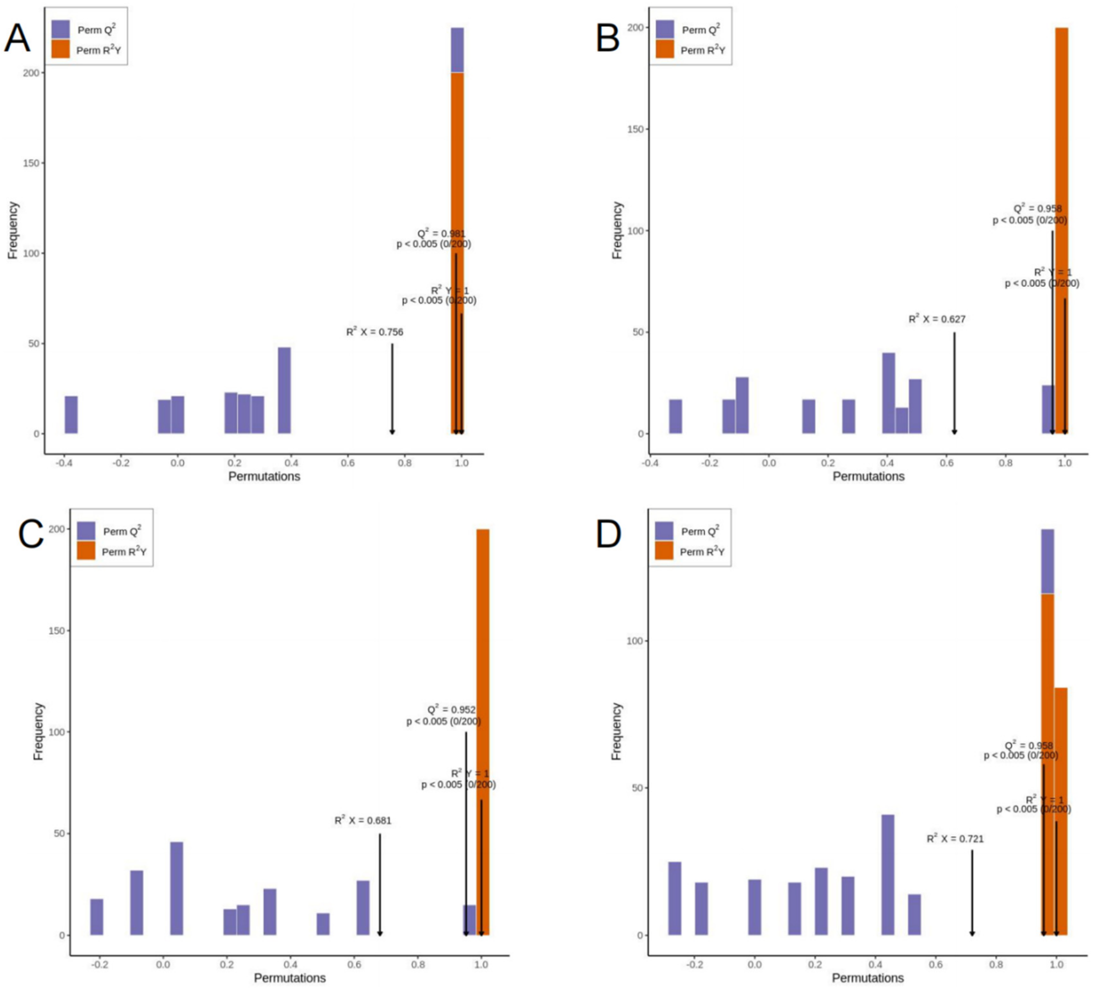

Figure S3. OPLS-DA permutation of GL vs. other production areas. A: GL_vs_CS_opls_permutation; B: GL_vs_JY_opls_permutation; C: GL_vs_MZ_opls_permutation; D: RX_vs_GL_opls_permutation. In general, the model is optimal when $p < 0.05$.

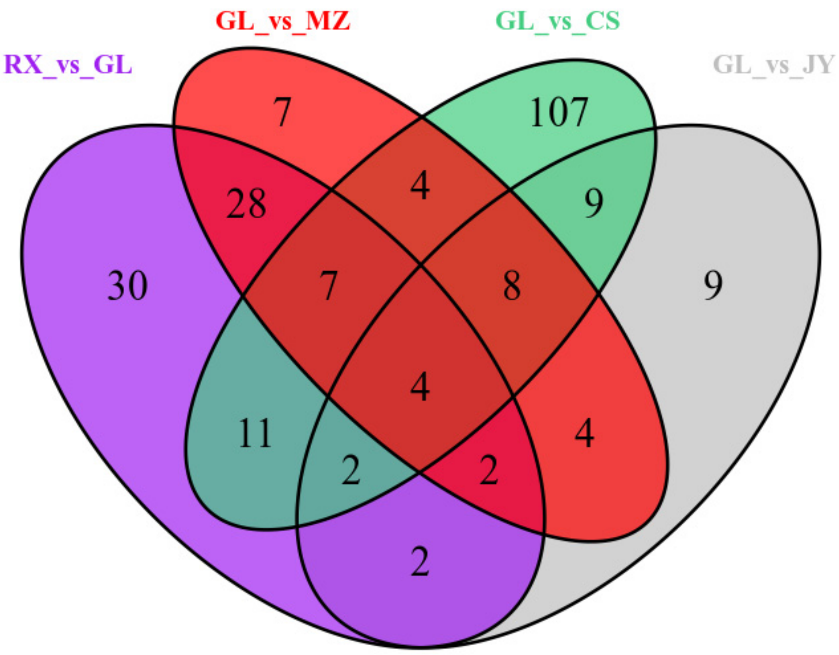

Figure S4. Comparison of GL with other appellations.
